# Supplementary material for: Competitive interactions between culturable bacteria are highly non-additive
Source: eLife. 2023 Feb 28;12:e83398. doi: 10.7554/eLife.83398 (PMC10072878; doi:10.7554/eLife.83398)
Supplement: Supplementary file 1. [file elife-83398-supp1.docx]

**Supplementary File 1a: Strains used in this study**

Rows highlighted with bold text represent the focal species, full phylogenetic data included in Data S1 file

| Nearest Species | Source | In Trios Subset |
| --- | --- | --- |
| *Microbacterium kyungheense* | This Study | Yes |
| *Chryseobacterium lactis* | This Study |  |
| *Chryseobacterium lactis* | This Study | Yes |
| *Chryseobacterium lactis* | This Study | Yes |
| *Chryseobacterium lactis* | This Study | Yes |
| *Chryseobacterium lactis* | This Study | Yes |
| *Chryseobacterium lactis* | This Study | Yes |
| *Chryseobacterium lactis* | This Study | Yes |
| *Chryseobacterium lactis* | This Study |  |
| *Chryseobacterium lathyri* | This Study | Yes |
| *Chryseobacterium lathyri* | This Study |  |
| *Flavobacterium banpakuense* | This Study | Yes |
| *Flavobacterium banpakuense* | This Study | Yes |
| *Flavobacterium ginsengiterrae* | This Study | Yes |
| *Bacillus albus* | This Study | Yes |
| *Bacillus cereus* | This Study | Yes |
| *Bacillus cereus* | Cytryn Lab |  |
| *Bacillus cereus* | Cytryn Lab |  |
| *Bacillus proteolyticus* | Cytryn Lab |  |
| *Bacillus proteolyticus* | Cytryn Lab |  |
| *Bacillus safensis* | This Study |  |
| *Bacillus safensis* | This Study |  |
| *Bacillus subtilis* | Cytryn Lab |  |
| *Bacillus thuringiensis* | Cytryn Lab |  |
| *Lysinibacillus macroides* | This Study | Yes |
| *Agrobacterium tumefaciens* | Kehe et al. |  |
| ***Buttiauxella izardii*** | **Kehe et al.** |  |
| *Buttiauxella izardii* | Kehe et al. | Yes |
| *Citrobacter braakii* | Kehe et al. |  |
| *Ewingella americana* | Kehe et al. | Yes |
| ***Ewingella americana*** | **Kehe et al.** |  |
| *Ewingella americana* | Kehe et al. |  |
| *Enterobacter aerogenes* | Kehe et al. |  |
| *Enterobacter cloacae* | Cytryn Lab | Yes |
| *Enterobacter cloacae* | Cytryn Lab |  |
| *Enterobacter cloacae* | Cytryn Lab |  |
| *Escherichia coli* | Kehe et al. | Yes |
| ***Escherichia coli*** | **Kehe et al.** |  |
| *Lelliottia amnigena* | Kehe et al. |  |
| *Pantoea agglomerans* | Kehe et al. |  |
| *Pantoea agglomerans* | Kehe et al. |  |
| ***Pantoea agglomerans*** | **Kehe et al.** |  |
| *Pantoea allii* | Kehe et al. |  |
| *Pantoea eucalypti* | Kehe et al. |  |
| *Pantoea vagans* | Kehe et al. |  |
| *Pseudomonas brassicacearum* | This Study |  |
| *Pseudomonas citronellolis* | This Study |  |
| *Pseudomonas corrugata* | This Study | Yes |
| *Pseudomonas helmanticensis* | Kehe et al. |  |
| *Pseudomonas koreensis* | Kehe et al. | Yes |
| *Pseudomonas koreensis* | This Study | Yes |
| *Pseudomonas koreensis* | This Study |  |
| *Pseudomonas monteilii* | This Study | Yes |
| *Pseudomonas monteilii* | This Study | Yes |
| *Pseudomonas mosselii* | This Study | Yes |
| *Pseudomonas plecoglossicida* | Kehe et al. |  |
| *Pseudomonas putida* | This Study | Yes |
| *Pseudomonas putida* | This Study | Yes |
| *Pseudomonas putida* | This Study | Yes |
| *Pseudomonas rhodesiae* | Kehe et al. |  |
| *Pseudomonas rhodesiae* | Kehe et al. |  |
| *Pseudomonas simiae* | This Study | Yes |
| *Pseudomonas viridiflava* | This Study |  |
| ***Raoultella planticola*** | **Kehe et al.** |  |
| ***Citrobacter freundii*** | **Kehe et al.** |  |
| *Citrobacter freundii* | Kehe et al. |  |
| *Comamonas odontotermitis* | This Study |  |
| *Serratia fonticola* | Kehe et al. |  |
| *Serratia fonticola* | Kehe et al. |  |
| *Serratia fonticola* | Kehe et al. |  |
| *Stenotrophomonas rhizophila* | This Study | Yes |
| *Variovorax guangxiensis* | This Study |  |
| *Raoultella planticola* | Kehe et al. | Yes |
| *Raoultella planticola* | Kehe et al. |  |
| *Raoultella planticola* | Kehe et al. |  |

**Supplementary File 1b: Carbon sources and antibiotics used in species profiling experiment**

| Carbon Source | Conc. | Antibiotic | Conc. |
| --- | --- | --- | --- |
| Glucose D+ | 1% w/v | Kanamycin | 50 μg/ml |
| D-Ribose | 1% w/v | Spectinomycin | 60 μg/ml |
| D-Xylose | 1% w/v | Amoxicillin | 50 μg/ml |
| D-Fructose | 1% w/v | Gentamicin | 20 μg/ml |
| D-Galactose | 1% w/v | Streptomycin | 100 μg/ml |
| Sucrose | 1% w/v | Trimethoprim | 50 μg/ml |
| D-Cellobiose | 1% w/v | Carbenicillin | 12.5 μg/ml |
| D-Lactose monohydrate | 1% w/v | Vancomycin | 50 μg/ml |
| D-Maltose monohydrate | 1% w/v | Rifampicin | 100 μg/ml |
| Sodium Citrate Dihydrate | 1% w/v | Chloramphenicol | 20 μg/ml |
| Disodium Succinate | 1% w/v | Ampicillin | 100 μg/ml |
| Sodium Acetate | 1% w/v |  |  |
| Sodium Pyruvate | 1% w/v |  |  |
| Glycerol | 1% v/v |  |  |
| D-Mannitol | 1% w/v |  |  |
| D-Sorbitol | 1% w/v |  |  |
| L-Serine | 1% w/v |  |  |
| L-Alanine | 1% w/v |  |  |
| L-Proline | 1% w/v |  |  |
| L-Lysine | 1% w/v |  |  |
